# Scripta Diffusa

**Published:** 1853-01

**Authors:** 


### ART. II. — *Scripta Diffusa.* By Smelfungus.

" Non fumum ex fulgore, sed ex fumo dare lucem cogitat."—*Ars Poetica Horat.*

" Scribendi recte sapere est et principium et fons."—*Ibid.*

Whether, had he lived long enough since, my friend Smelfungus would have been a Broussaist or Brunonian, a Rushite or Tullyite, no man knoweth. My friend's qualities — good and bad — are all positive — noncommittalism was never his forte.

Therefore Smelfungus is no *expectant.* It is one of his peculiarities, that, sitting in his cobwebed and smoke-stained office, he builds hypothetical castles in the air, and fights doughty battles with imaginary antagonists. Late in the lonely night he grows warm in argument, and waves his long right arm up and down after the manner of a pump-handle, in solitary dignity. Hear him now!

"This expectant theory is the offspring of scepticism! The medical mind is like any other mind. It would fain have certainty. As in intellectual philosophy; amid conflicting theories, where twenty rival truths stand up together to confound the page of inquiry; bewildered among deism and atheism, revelation and geology, spiritualism and materialism, idealism and eclecticism; but between Bossuet and Spinoza, Locke and Kant, the mind finds refuge in unbelief.

"Unable to reconcile all these, it turns to blind Chance as its only Creator; and in the very utmost stretch of pride of Reason, it bows itself to the disgrace of annihilation.

"Kindred causes make the expectant. To him two great and conflicting facts present themselves. One, the now acknowledged power of the *vis medicatrix naturae* in conducting disease to a safe termination: the other, the well-known influence of medicine. 'If,' he argues, 'twenty cases of pneumonia get well without treatment, and twenty get well with treatment, where has the physician the advantage over nature?'

"But, my dear Expectant," says Smelfungus, and up goes his pump-handle arm, as if to send the bucket to the lowest depth of the well of intellect; this proves nothing." It may be that every one of Dame Nature's cases would have been the better for antimony and blistering. Say nothing of the slovenly manner in which she performs her cures, and it is still evident that medical aid is efficient. Here you have an armament of cures; mighty weapons wrung from the bowels of the earth, the depths of the sea, or the surface of the dry land. With them you can change and modify the secretions, you can check and turn the current of the circulation, you can prostrate lusty health itself in death — are you then powerless in disease? Nature cures her patients — so do you. Do two affirmatives make a negative?

"Sir, if with all these powerful agents of the materia medica you have no control over disease, then are you a weak sister! You would be '*interpres naturae et adjutor;*' be also her governor!

"It is said that 'incredulity is the first element of a philosophical mind.' It may the first but there is a higher one — *Faith!* This expectant theory savors of Pantheism. If it be true, Homœopathy is justifiable. It *is* a Pantheon, to which every idolatrous quack may bring his God!

"But I, Smelfungus, will yet believe that in mercury we have the mighty changer of secretions, in quinia the wonderful controller of disordered nervous action, and in opium the kind nepenthe for physical suffering."

So much from Smelfungus on the expectant theory. I think were the truth known, that our friend intended in the beginning of his cogitations to think of something else. So let all this blast at the expectants go for nothing, while he resumes his broken chain of thought, and talks about blood poisons.

"I never was a solidist. All things solid are dry, and all things dry are dead. Juiciness is the *sine qua non* of vitality. But the most sanguine humoralist of former days would have stared, could he have known the extent to which humoralism is now carried. It does not stop with the contagious exanthemata, but runs on through the whole nosology. Not even nervous disease is excepted. *I* do n't except it. The other day I treated a case of obstinate and persistent neuralgia with, what think you? It had previously resisted quinine, morphine, zinc, valerian, &c. Calomel, and that alone was the remedy, and was entirely successful in a few hours, and made a *permanent* cure. The rationale is simple. Clay-colored stools, and slight icterus accompanied the neuralgia. Now bile detained in the circulation is a blood poison, acting upon the nervous system, usually producing somnolency and dull headache. But these blood poisons act not always alike. Chloroform sometimes confines its effects to the motor tract of the spinal cord, giving no immunity from suffering. So bile may change the locality of its effect, and attacking the sensative tract of the spinal cord, may cause neuralgic pain. Once upon a time, Smelfungus himself had neuralgia of the fifth pair. A certain distinguished professor traced a sinister relationship between it and an erysipelas then prevailing, and prescribed (*ex pede gnosce Herculem*) castor oil and turpentine as a 'depurant cathartic.' I have never forgiven him for that dose of unmitigated nastiness. It acted as an emetic merely. Another professor came; and when blue pill had done its perfect work, I slept a sleep the memory whereof is like a pleasant dream.

"In the last number of Braithwaite is an article 'on the blood origin of a certain form of palsy, by Dr. Hamilton Kinglake.' Here was a palsy, creeping gradually over the patient, until it was, to all appearance, likely to terminate fatally. But Dr. Kinglake followed out the indications presented to him, until he (*Dr. Kinglake* and *not* Dame Nature) effected a cure. I think his hypothesis is justifiable. He argues that the retention of urea in the blood caused the palsy, and the evident good effect of colchicum in the case gives probability to the idea. It is well known, and actual experiment has proved, that colchicum does increase to a notable degree the quantity of urea eliminated by the kidneys. Carry out the suggestions afforded by this case and even you, oh Expectant! tossing upon the ocean of doubt and sceptcism, may as you rise anon from the trough of the sea, catch a bright glimpse of the load-star of certainty in medinine.

"Feeble vision is not total darkness. The alembic of the chemist is solving many a weary problem, and shall yet place in our hands the divining rod which shall point us to the hidden springs of disease."

As the most intimate friend of Dr. Smelfungus, it is my wish to make him shine in these papers. So I have urged him to take a text and stick to it. But he wont do it, and seems determined to prove Sidney Smith's assertion, that no man can, for two consecutive minutes, confine his thoughts to a single subject. If he does scatter his ideas like a watering-pot, let us hope that some feeble plant of wisdom may benefit by his aspersions — some "herb of grace" grow stronger. The urea has got into his brain now — for conscience sake let him urinate:

"I have got a patient who never had hysteria. She has palpitation — pain in left shoulder and left cheek-bone, numbness in the left arm, and flying pains in her wrists, ankles, and other locations. When these symptoms are worst, she makes many pints of limpid urine daily; this urine very deficient in urea. I believe nobody has ever attempted to account for the liberal outpouring of colorless urine which accompanies hysteria, epilepsy, and not unfrequently neuralgia. Would it be unphilosophical to suppose" (the reader must already have perceived that Smelfungus has a holy horror of hypothesis) "that this symptom may stand rather in the relation of a cause, than of an effect? and that colchicum may yet become an important remedy in nervous disease? And now I'll tell you something queer: this same patient was confined last spring at seven months, with a scrawny pair of twins. Her pains commenced in the left ankle, and there remained from 9, A. M. till 10, P. M., the os uteri meanwhile dilating as regularly as need be, without pain in the uterus. At 10 o'clock, P. M., the pains commenced in the uterus, the os being then well dilated, and delivery was completed in an hour. Furthermore, she had her afterpains in her ankle.

"What has this to do with blood poisons? It has a bearing; had you the eye to see it? If this patient had pure blood in her veins, think you she would have suffered those strange anomalies? That her blood was impure is proved by the existence of nursing sore mouth, for some time before and after confinement.

"Of late years sundry observers have noticed sudden deaths occurring in eruptive diseases, and attributable to subarachnoid effusion. The poison of scarlatina seems the more common cause of this, but it is also a sequel to other diseases. I have witnessed it in four cases. One was a child, convalescing from an apparently mild form of measles. In the other three cases neuralgia was a prodrome. In one of them I was unable to trace blood-poison, though as I only saw it *in articulo mortis* this may have no bearing. But in the two remaining cases — in one of them from nursing sore mouth — in the other from chlorotic anæmia — there was an unmistakable deterioration of the blood. In the latter case, by putting together this blood disease and a neuralgia of the back and side of the head, I was enabled, six weeks before death, to anticipate and warn the friends of the probability of subarachnoid effusion. Five minutes before her death, she complained of pain in the occiput and back of her neck, and requested her husband to rub her neck. While doing so, he found her insensible, with interrupted respiration, and she immediately died. It is not improbable that we might oftener be prepared for these events, were we to watch closer for faults in the rhythm of respiration, connected with neuralgic pain. And it is in blood diseases that this accident will most frequently occur."

It is curious that Smelfungus has kept away from the subject of malaria so long in this discussion. But it is growing late, and the reader must be deprived of his argument thereon. But before we part he grows prophetic, and with his long nose elevated, he foretells the advent of a day, when by the aid of chemistry and an enlightened physiology, the physician shall prescribe, with an unfailing certainty, for the elimination of morbid matter from the blood, or the supply of deficient healthy constituents.

However unwilling to cast a shade upon the enthusiasm of my worthy friend, I could not resist the temptation to quote to him the pithy lines of Hudibras:

> "The wind that's in the belly pent,
> Is but a (*something unmentionable*) if downward sent,
> But if perchance it upward rise,
> *It vents itself in prophecies!*"

Smelfungus dashes his pipe upon the floor, and so breaks up our lengthy *sederunt.*

